# Incentives conditioned on tenofovir levels to support PrEP adherence among young South African women: a randomized trial

**DOI:** 10.1002/jia2.25636

**Published:** 2020-11-28

**Authors:** Connie L Celum, Katherine Gill, Jennifer F Morton, Gabrielle Stein, Laura Myers, Katherine K Thomas, Margaret McConnell, Ariane van der Straten, Jared M Baeten, Menna Duyver, Eve Mendel, Keshani Naidoo, Jacqui Dallimore, Lubbe Wiesner, Linda‐Gail Bekker

**Affiliations:** ^1^ Department of Global Health University of Washington Seattle WA USA; ^2^ Department of Medicine University of Washington Seattle WA USA; ^3^ Department of Epidemiology University of Washington Seattle WA USA; ^4^ The Desmond Tutu HIV Centre University of Cape Town Cape Town South Africa; ^5^ Chan School of Public Health Harvard University Boston MA USA; ^6^ Women’s Global Health Imperative RTI International Berkeley CA USA; ^7^ Department of Pharmacology University of Cape Town Cape Town South Africa; ^8^ Center for AIDS Prevention Studies Department of Medicine University of California San Francisco CA USA

**Keywords:** HIV pre‐exposure prophylaxis, adherence, drug level feedback, incentives, young women, Africa

## Abstract

**Introduction:**

HIV incidence remains high among African adolescent girls and young women (AGYW), who would benefit from pre‐exposure prophylaxis (PrEP). Strategies to increase PrEP adherence and persistence need to be evaluated in African AGY, including incentives conditional on high adherence.

**Methods:**

The 3Ps for Prevention Study was a 12‐month prospective cohort of 200 women ages 16 to 25 initiating PrEP in South Africa from 2017 to 2018. Participants received retrospective feedback about drug levels at Months 1, 2 and 3; half was randomized to receive a 200 Rand shopping voucher ($13 US) at Months 2, 3 and 4, conditioned on high intracellular tenofovir diphosphate (TFV‐DP) levels in dried blood spots (≥500 fmol/punch at Month 1, ≥700 fmol/punch at Months 2 and 3). The primary analysis was intention‐to‐treat, comparing the proportion with high PrEP adherence (≥700 fmol/punch) at Month 3 by randomized group, based on 100% efficacy among men who have sex with men.

**Results:**

Median age of the 200 women was 19 years (interquartile range [IQR] 17, 21); 86% had a primary sexual partner. At Month 3, the mean TFV‐DP level was 822 fmol/punch (SD 522) in the incentive group and 689 fmol/punch (SD 546) in the control group (*p* = 0.11). Forty‐five (56%) of 85 women in the incentive group and 35 (41%) of 85 women in the control group had TFV‐DP levels ≥700 fmol/punch (RR 1.35; 95% CI 0.98, 1.86; *p* = 0.067), which declined to 8% and 5% in the incentive and control groups at Month 12 (no significant difference by arm). 44% refilled PrEP without gaps, 14% had a gap of ≥3 weeks in coverage subsequently restarted PrEP and 54% accepted at the final dispensing visit at Month 9. No new HIV infections were observed after PrEP initiation.

**Conclusions:**

Among South African AGYW initiating PrEP, drug levels indicated high PrEP adherence in almost half of women at Month 3, with a non‐statistically significant higher proportion with high adherence among those in the incentive group. Over half persisted with the 12‐month PrEP programme although high adherence declined after Month 3. Strategies to support PrEP adherence and persistence and longer‐acting PrEP formulations are needed.

## 1 INTRODUCTION

Adolescent girls and young women (AGYW) in sub‐Saharan Africa account for approximately 25% of new HIV infections globally. Recent HIV prevention trials have documented high HIV incidence despite monthly counselling and prevention services [1, 2, 3]. Tenofovir‐based pre‐exposure prophylaxis (PrEP) confers >90% protection against HIV when taken with high adherence [4, 5, 6]. However, open‐label studies of PrEP among African AGYW have demonstrated substantial drop‐off in the first few months, which may reflect low awareness about PrEP, changing risk perception and motivation and initial side effects with PrEP. Persistence with contraception has also been difficult for African AGYW with 40% to 65% persistence at one year for hormonal contraceptive methods [7]. PrEP persistence may be more challenging than contraception given the lack of familiarity with antiretrovirals for prevention, stigma from PrEP being misperceived as taking antiretrovirals for treatment [8], and changing partnerships and perceived prevention need [9].

Qualitative research from two placebo‐controlled PrEP efficacy trials among African AGYW (i.e. VOICE and FEM‐PrEP) among African AGYW indicated reasons for low PrEP use including receiving an investigational drug of unknown efficacy or a placebo, fear of side effects, low research literacy, the need for social support and fear of their partner’s reactions [10, 11, 12]. In a substudy after the VOICE trial was unblinded, women in the active arms who received their drug levels reported that feedback about their adherence would motivate use and more honest discussions about challenges they faced in PrEP use [12]. In trials of oral and topical PrEP products that provided objective adherence feedback to participants, the acceptability has been high [13, 14, 15, 16].

Behavioural economics provides a framework for understanding and designing interventions to overcome behavioural biases, such as present bias, optimism bias and lack of saliency, which may undermine adoption and persistence with prevention behaviours [17, 18]. Drug level feedback and incentives that are conditioned on high adherence to PrEP may be particularly pertinent to young African women who may find the new behaviour of pill‐taking for HIV prevention difficult, given their developmental stage and challenges with inhibitory control [19, 20, 21, 22]. Conditional incentives have been shown to increase uptake of some HIV prevention behaviours, including HIV testing [23, 24, 25] and voluntary medical male circumcision [26, 27]. The potential for incentives conditioned on high drug levels to motivate PrEP adherence and overcome initial barriers to PrEP adherence and persistence (e.g. concern about side effects) has not been evaluated among young African women.

Given the high HIV incidence among South African AGYW, it is important to evaluate which interventions are effective in improving the effective use of PrEP through increased adherence and persistence. The 3Ps for Prevention Study (Partners, Perception and Pills) was designed to assess PrEP adherence execution (how well young women took PrEP as measured by objective drug levels) and persistence (how long they continued to attend visits and receive PrEP) in a prospective cohort of South Africa AGYW receiving drug level feedback. We conducted a randomized evaluation of a behavioural economics intervention of incentives conditioned on intracellular tenofovir drug levels in the first three months of PrEP use in the 3Ps for Prevention Study, comparing PrEP adherence levels of those receiving conditional incentives to those receiving drug level feedback alone.

## 2 METHODS

### 2.1 Study population

Between March 2017 and May 2018, HIV‐negative women ages 16 to 25 were recruited for the 3P study in a high‐density, low‐income periurban township on the outskirts of Cape Town, South Africa (ClinicalTrials.gov NCT03142256). Women were eligible if they had vaginal or anal sex in the month prior to screening or were planning on being sexually active in the next three months, willing to take PrEP, not pregnant, and had normal renal function (creatinine clearance ≥60 mL/min).

### 2.2 Randomization

At enrolment, participants were randomized using REDCap in a 1:1 ratio to the standard of care adherence group, which received structured adherence counselling at each study visit and drug level feedback at Months 2, 3 and 4, or to the enhanced adherence group, which also received an incentive conditioned on high adherence based on drug levels at Months 2, 3 and 4. At the Month 4 time point, drug level feedback occurred by phone or home visit, and provided at their Month 6 visit, if they could not be reached.

### 2.3 Study procedures

**Figure 1:**
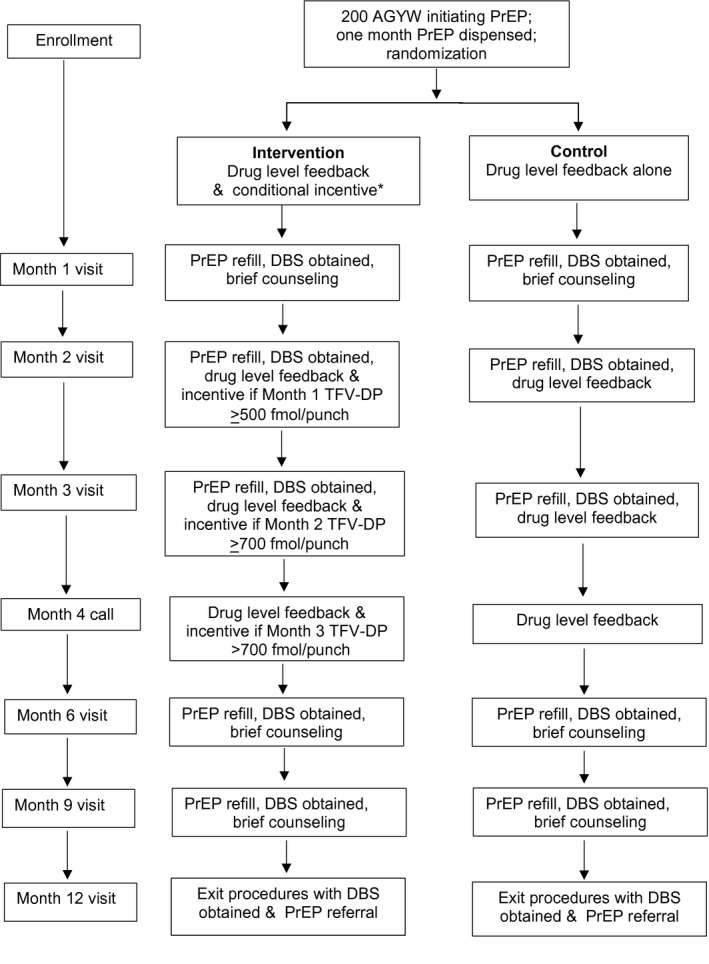
Study design. A bottle with 30 tablets for a 30 day PrEP supply was provided at enrollment, Months 1 and 2. At Months 3, 6, and 9, three bottles of 30 tablets were provided for a three month refill, Drug level feedback used semiquantitative levels and counseling messages based on high protection (TFV‐DP >500 fmol/punch for the month one sample and >700 fmol/punch for subsequent time points). The low protection group included the range from detectable (16 fmol/punch lower limit of detection to <500 fmol/punch at month one and <700 fmol/punch at months two through nine). A bottle with 30 tablets for a 30 day PrEP supply was provided at enrollment, Months 1 and 2. At Months 3, 6, and 9, three bottles of 30 tablets were provided for a three month refill, Drug level feedback used semiquantitative levels and counseling messages based on high protection (TFV‐DP >500 fmol/punch for the month one sample and >700 fmol/punch for subsequent time points). The low protection group included the range from detectable (16 fmol/punch lower limit of detection to <500 fmol/punch at month one and <700 fmol/punch at months two through nine).

Study visits were at Months 1, 2, 3, 6, 9 and 12 with HIV and pregnancy testing at every visit (Figure 1). Dried blood spots (DBS) were obtained at Months 1, 2 and 3 to measure intracellular tenofovir diphosphate (TFV‐DP) levels, which have an estimated 17‐day half‐life in red blood cells and provide an estimate of cumulative dosing in the prior four to six weeks [28].

Women in both groups received drug level feedback about their DBS results from the prior visit at Month 2 and 3 visits and by phone call or home visit at Month 4, using a semi‐quantitative image of a wireless signal with high, medium and low categories of adherence (Figure 1). High adherence was defined using a threshold of TFV‐DP ≥ 500 fmol/punch) in the Month 1 DBS sample, given that steady‐state levels of intracellular TFV‐DP are not reached until six weeks, and ≥700 fmol/punch in the Month 2 and 3 DBS samples. The 700 fmol/punch threshold correlates with taking an average of four doses per week in the prior four to six weeks in a study of directly observed dosing in a male US population [28], which was associated with 100% PrEP effectiveness in men who have sex with men [29]. Medium adherence was defined as an average of one to three doses per week in the prior month (TFV‐DP ≥ 250 to 499 fmol/punch at Month 1 and ≥350 to 699 fmol/punch at Month 2 and beyond) and low adherence as above the limit of detection of 16 fmol/punch to 250 fmol/punch at Month 1 and 350 fmol/punch at Month 2 and beyond. In the intervention group, for each visit that a woman achieved high adherence, she received a shopping voucher with the value of 200 South African Rand (equivalent to US $13) at the next clinic visit.

PrEP was withheld from women who became pregnant, consistent with South African guidelines at the time, those whose creatinine clearance decreased to <60 mL/min, or who had a positive rapid HIV test. Women could restart PrEP after pregnancy and when their creatinine clearance returned to ≥60 mL/min, or if additional HIV testing with RNA confirmation determined that they were not HIV infected.

### 2.4 Laboratory methods

Creatinine clearance was estimated based on serum creatinine measurements using the Cockroft Gault equation. Testing for *Neisseria gonorrhoeae* (GC) and *Chlamydia trachomatis* (CT) was by nucleic acid amplification (GeneXpert®, Cepheid, Sunnyvale, CA), and *Trichomonas vaginalis* (TV) by rapid test (OSOM® Trichomonas Test, Sekisui Diagnostics, Burlington MA). HIV status was determined by a point of care fourth‐generation rapid HIV test (Determine Abbott, Abbott Park, IL USA) followed by the Trinity Unigold test, and if either was reactive, an instrumented fourth‐generation antigen/antibody immunoassay (Architect Abbott Combo®, Abbott Laboratories). If the fourth‐generation instrumented test was positive, RNA testing was performed to differentiate acute infection from a false‐positive antigen/antibody test.

For drug level feedback, intracellular TFV‐DP levels in DBS were measured at the University of Cape Town Division of Clinical Pharmacology Laboratory, which developed and validated an indirect method for the quantification of tenofovir diphosphate in 50 µL human DBS, in collaboration with the University of Colorado Pharmacology laboratory, which developed the assay and determined thresholds with directly observed dosing [28].

### 2.5 Ethical review

The study protocol was approved by the Health Science ethics review committee at the University of Cape Town. All participants provided written informed consent in English or Xhosa. A parental consent waiver was granted for participants ages 16 to 17.

### 2.6 Endpoints

The primary endpoint was TFV‐DP fmol/punch ≥700 fmol/punch in DBS at Month 3 after PrEP initiation, compared by randomized group, in order to determine whether the conditional incentives had an effect on adherence while the interventions were provided. We chose the ≥700 fmol/punch reflecting an average of four pills per week as a realistic and achievable goal which was associated with 100% protection among men who have sex with men [29]. We also analysed TFV‐DP fmol/punch levels at Month 3 as a continuous variable, by randomized group. Secondary endpoints were TFV‐DP levels at Months 6 and 12 to assess the durability of the intervention on PrEP adherence, overall and by randomized group.

### 2.7 Statistical analysis

The sample size of 200 and 10% loss to follow‐up was estimated to provide 80% power to detect the difference between 40% with high adherence at Month 3 in the control group versus 62% in the intervention group. The 22% difference in effect size was in part determined by a relatively large improvement in adherence needed to justify additional costs and logistics of providing conditional incentives.

Descriptive statistics of data was provided to describe retention, PrEP acceptance, persistence with the PrEP programme and discontinuations; retention was retention to study visits, and was defined as attending a study visit within the study visit window; PrEP acceptance was defined as obtaining a PrEP refill at which it was offered (Months 1, 2, 3, 6 and 9). Persistence with the PrEP programme was defined as being dispensed PrEP at the final PrEP dispensing visit at 9 months. PrEP discontinuation was defined as a missed refill due to a missed visit, or ≥21 days not taking PrEP. Participants could have one or more discontinuations during follow‐up while being defined as persisting with the programme if they subsequently restarted. Retention was defined based on attending the study visit within the visit window.

The primary analysis was an intention‐to‐treat comparison of the proportion with TFV‐DP ≥ 700 fmol/punch at Month 3 by randomized group, estimated as a relative risk (RR) from a modified Poisson regression model with a robust error variance [30]. Because participants not attending the Month 3 visit were thought likely to have stopped taking PrEP, and therefore would have had low‐drug concentration if a DBS had been obtained, we also performed a secondary analysis in which missing DBS results (either due to missing the study visit, or not providing DBS at the visit) were imputed as having low adherence. A pre‐specified subgroup analysis evaluated effect modification by age (dichotomized as < or ≥20 years), adding age and an interaction term between age and randomized group in the primary analysis. The durability of an effect of the conditional incentives was examined by repeating the primary analysis, but using TFV‐DP results at Month 6 (for mid‐term duration) and Month 12 (for longer‐term duration). To describe adherence more completely, outcomes of any detectable TFV‐DP, and medium adherence of TFV‐DP ≥ 350 to 699 (≥250 to 499 for month 1) are also presented. The effect of the intervention on the continuous adherence outcome TFV‐DP levels at Month 3 was estimated as the difference in mean adherence levels between those in the intervention versus control group, using a linear regression model with the outcome of continuous DBS TFV‐DP level, and randomized group as the primary predictor. Statistical analyses used SAS 9.4.

## 3 RESULTS

### 3.1 Cohort characteristics

The median age of the 200 women enrolled in the study was 19 years (range or IQR, 17 to 21), and 77% had completed some secondary school education or higher (Table 1). At enrolment, 86% reported a primary sexual partner with a median partnership duration of 12 months (IQR 6, 36 months); 34 (20%) reported a partner who was at least five years older, 109 (55%) did not know if their partner had other partners and 33 (17%) reported he had additional partners. Most women reported their partner was HIV‐uninfected (110, 55%) or did not know their partner’s status (89, 45%), with only one (0.5%) reported having an HIV‐infected partner. Baseline prevalence of curable sexually transmitted infections (STIs) was 32%, with 25% *C. trachomatis*, 11% *N. gonorroheae* and 6% *T. vaginalis*. Only five women (3%) reported STI symptoms.

**Table 1:** Baseline demographic and behavioural characteristics

Characteristic N(%) or median (IQR)	Incentive N = 101	Control N = 99	Total N = 200
Demographics			
Age, median (years)	19 (17.0, 21.0)	19 (17.0, 21.0)	19 (17.0, 21.0)
Education			
Primary or some secondary school	52 (61.4%)	61 (62.3%)	113 (61.8%)
Completed secondary school or higher	39 (38.7%)	35 (34.7%)	60 (30.2%)
Partnership characteristics			
Has a primary sexual partner	82 (81.2%)	90 (90.9%)	172 (86.0%)
Primary partner is ≥ 5 years older than participant	12 (14.6%)	22 (24.4%)	34 (19.8%)
Primary partnership duration (months)	12 (6.0, 36.0)	12 (6.0, 24.0)	12 (6.0, 36.0)
Number of sex partners, past three months			
0	17 (16.8%)	10 (10.1%)	27 (13.5%)
1	78 (77.2%)	83 (83.8%)	161 (80.5%)
≥2	6 (5.9%)	6 (6.1%)	12 (6.0%)
Partner has other sex partners			
Yes	17 (16.8%)	16 (16.2%)	33 (16.5%)
No	29 (28.7%)	29 (29.3%)	58 (29.0%)
Not sure	55 (54.5%)	54 (54.5%)	109 (54.5%)
Partner HIV status			
HIV negative	60 (59.4%)	50 (50.5%)	110 (55.0%)
HIV positive	0 (0%)	1 (1.0%)	1 (0.5%)
Participant does not know	41 (40.6%)	48 (48.5%)	89 (44.5%)
Intimate Partner Violence in past year	20 (19.8%)	17 (17.3%)	37 (18.6%)
Transactional sex^a^	2 (2.0%)	3 (3.0%)	5 (2.5%)
Contraceptive use, Sexual and Alcohol use behaviours			
Contraceptive Method (multiple choices allowed)	100	98	198
None	1 (1.0%)	0 (0%)	1 (0.5%)
Birth control pills	7 (6.9%)	9 (9.1%)	16 (8.0%)
Injectable	73 (72.3%)	68 (68.7%)	141 (70.5%)
Implant	14 (13.9%)	12 (12.1%)	26 (13.0%)
IUD	0 (0%)	2 (2.0%)	2 (1.0%)
Male condoms	9 (8.9%)	9 (9.1%)	18 (9.0%)
Sexually active, prior 30 days	78 (77.2%)	74 (74.7%)	152 (76.0%)
Condom use, prior 30 days	78	74	152
Never	15 (19.2%)	18 (24.3%)	33 (21.7%)
Rarely or sometimes	32 (41.0%)	38 (51.4%)	2 (46.0%)
Often	5 (6.4%)	2 (2.7%)	7 (4.6%)
Always	26 (33.3%)	16 (21.6%)	42 (27.6%)
Alcohol use, prior three months			
Never	31 (30.7%)	27 (27.3%)	58 (29.0%)
Monthly or less frequently	59 (58.4%)	56 (56.6%)	115 (57.5%)
Weekly	10 (9.9%)	16 (16.2%)	26 (13.0%)
STI testing^b^			
Trichomonas	8 (7.9%)	4 (4.0%)	12 (6.0%)
Chlamydia	25 (25.0%)	24 (24.2%)	49 (24.6%)
Gonorrhoea	11 (11.0%)	10 (10.1%)	21 (10.6%)
Any STI (trichomonas, chlamydia and/or gonorrhoea)	34 (33.7%)	30 (30.3%)	64 (32.0%)
PrEP disclosure plans			
Disclosed plan to take PrEP	92 (91.1%)	90 (90.9%)	182 (91.0%)

^a^ Transactional sex is defined as having sex with a man because he provided her with or she expected he would provide her with food, clothes, cosmetics, items for children, transportation, cash, school fees, mobile phone air time and other items

^b^ Testing for *Neisseria gonorrhoeae* and *Chlamydia trachomatis* was by nucleic acid amplification (GenXpert™) and *Trichomonas vaginalis* by the rapid OSOM™ test.

### 3.2 Randomized groups and retention to study visits

**Figure 2:**
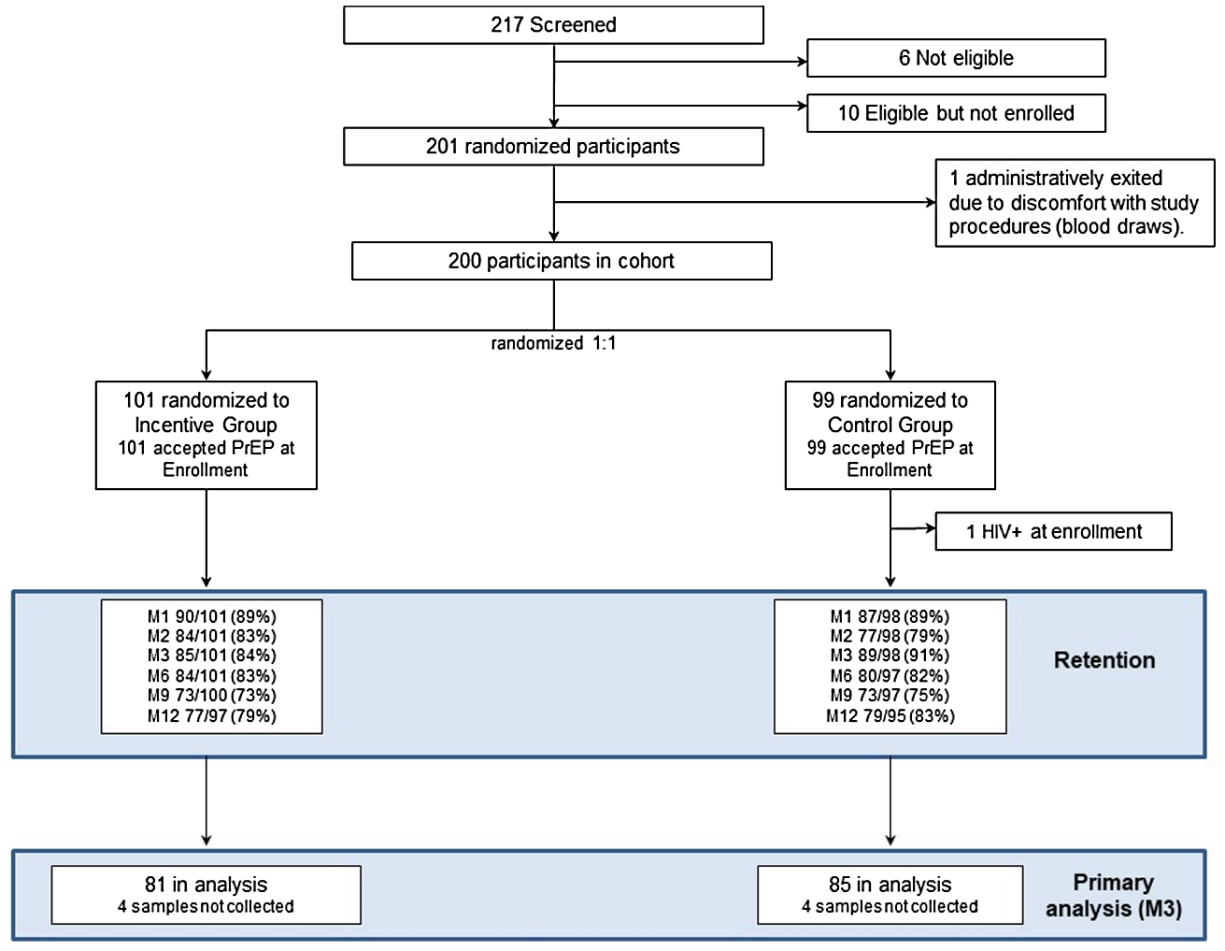
CONSORT diagram. Participant flow of women screened, enrolled and randomized in the 3P study. One woman was determined to be HIV‐infected at enrollment after her one month sample showed HIV‐1 antibodies; back testing of her enrollment sample which was positive for HIV RNA. The analysis sample includes 167 women at the month 3 visit who had a dried blood spot sample for tenofovir diphosphate levels for the primary three month adherence outcome.

All women were dispensed PrEP at enrolment, of whom 101 were randomized to receive incentives conditioned on drug levels, and 99 were randomized to receive drug level feedback alone, without incentives (Figure 2). Study retention across visits was 73% to 89% in the conditional incentive group, and 75% to 89% in the control group, with 79% and 83% retention at 12 months in the conditional incentive and control groups respectively.

### 3.3 Persistence with the PrEP programme, timing and reasons for PrEP discontinuation

**Figure 3:**
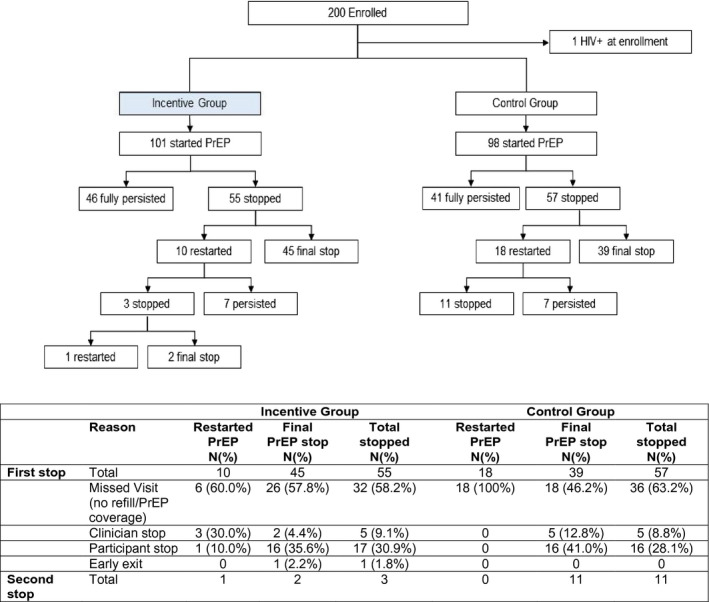
PrEP Discontinuation by randomized group. PrEP discontinuation’ was defined as missed refill due to missed visit, or ≥21 days not taking PrEP as documented in discontinuation form. Women who missed a refill but had a subsequent visit where they accepted PrEP were considered to have restarted PrEP. If a participant missed all follow‐up visits, the visit month of discontinuation is the first visit month missed. ‘Fully persisted with PrEP’ is defined as having had enough PrEP to cover through the Month 12 exit visit.

PrEP acceptance rates were high through Month 3: 99% at Month 1, 97% at Month 2 and 94% at Month 3 (Table 2). After Month 3, visits became quarterly, and PrEP acceptance declined to 79% of women in the incentive group and 90% in the control group at Month 6, and 76% and 73%, respectively, at Month 9. During follow‐up, 87 (44%) accepted PrEP refills throughout Month 9 and had no discontinuation, and an additional 14% of women restarted PrEP after discontinuing (Figure 3). One‐year persistence with the PrEP programme was 54%; 102 of 189 participants still eligible for PrEP at the final dispensing visit at Month 9 accepted PrEP, including as not persisting those who did not attend the 9‐month visit. Temporary PrEP discontinuations were largely due to missed visits (Figure 3). Among those who were retained in the 3P study, 70% of women who discontinued PrEP reported it was their preference to stop PrEP and 30% due to side effects.

**Table 2:** PrEP acceptance^a^ by randomized group and month

Study month	N accepted^a^ PrEP/ N eligible at visit (%)		
	Incentive	Control	Total
Enrolment	101/101 (100%)	98/98 (100%)	199/199 (100%)
Month 1	87/89 (97%)	86/86 (100%)	173/175 (99%)
Month 2	80/83 (96%)	73/74 (99%)	153/157 (97%)
Month 3	77/84 (92%)	82/86 (95%)	159/170 (94%)
Month 6	65/82 (79%)	68/76 (90%)	133/158 (84%)
Month 9	54/71 (76%)	48/66 (73%)	102/137 (74%)
Month 12	Exit visit	N/A	N/A

^a^ PrEP acceptance was defined as obtaining a PrEP refill at which it was offered (Months 1, 2, 3, 6 and 9).

### 3.4 PrEP adherence by randomized group

Of the 494 DBS results during the first three months, all but five had TFV‐DP detected indicating a very high proportion of women used PrEP in the first three months. Self‐reported adherence at Month 1 did not discriminate well between categories of observed intracellular TFV‐DP levels in the same time period; one of five women with undetectable TFV‐DP levels reported having poor or fair adherence, excellent adherence was reported by 11 (17%) of those in the intermediate adherence group by TFV‐DP, and very similar proportions in both intermediate and high adherence groups by TFV‐DP reported having either good (19/64 and 32/106, 30% of each) or very good (23/64 and 38/106, 36% of each) adherence.

For the primary outcome, DBS results were available for 166 participants at Month 3 (Figure 2); the proportion with TFV‐DP levels ≥700 fmol/punch in the incentive group was 56% (45/81) compared to 41% (35/85) in the control group. In the intention‐to‐treat (ITT) analysis, there was a non‐statistically significant trend of high adherence higher in the incentive arm (RR = 1.35; 95% CI 0.98, 1.86, *p* = 0.067; Table 3). DBS results were missing at Month 3 from 16 participants in the intervention and 10 in the control group who did not attend that visit, and four women in each group who attended the visit but a DBS specimen was not obtained (Figure 2). Secondary analysis interpreting missing DBS values as non‐adherence showed a similar trend, with high adherence among 45% (45/101) in the incentive versus 36% (35/98) in the control group (RR = 1.25; 95% CI 0.89, 1.76, *p* = 0.21). When TFV‐DP levels at Month 3 were analysed as a continuous variable, the mean TFV‐DP level in the incentive group was 822 fmol/punch (standard deviation, SD 522) and in the control group was 689 (SD 546; *p* = 0.11). A subgroup analysis of age (<20 and ≥20 years old) in the intention‐to‐treat comparison showed no effect modification with high adherence at Month 3 (RR = 1.30; 95% CI 0.84, 2.02) among AGYW < 20 years compared to RR = 1.42 (95% CI 0.90, 2.24 among those ≥21 years, *p* = 0.79).

**Table 3:** PrEP adherence, defined as high and medium adherence, any and average use measured by intracellular tenofovir‐diphosphate levels at Months 1, 2, 3, 6 and 12, by study randomized group

	High adherence by TFV‐DP ≥700 fmol/punch^a^				Medium adherence by TFV‐DP ≥350 to 699 fmol/punch^b^				TFV‐DP Detected (> 16.6 fmol/punch)				TFV‐DP fmol/punch Median (IQR) if detected	
	Incentive N (%)	Control N (%)	Relative risk (95% CI)	*p*‐value	Incentive N (%)	Control N (%)	Relative risk (95% CI)	*p*‐value	Incentive N (%)	Control N (%)	Relative risk (95% CI)	*p*‐value	Incentive	Control
Month 1	53/87 (60.9%)	53/85 (62.4%)	0.98 (0.77, 1.24)	0.85	75/87 (86.2%)	74/85 (87.1%)	0.99 (0.88, 1.11)	0.87	86/87 (98.9%)	85/85 (100%)	0.99 (0.97, 1.01)	0.32	646 (375, 934)	610 (421, 811)
Month 2	44/81 (54.3%)	34/75 (45.3%)	1.20 (0.87, 1.65)	0.27	65/81 (80.3%)	62/75 (82.7%)	0.97 (0.84, 1.13)	0.70	81/81 (100%)	75/75 (100%)	–	–	792 (413, 1099)	675 (384, 930)
Month 3														
*ITT*	***45/81 (55.5%)***	***35/85 (41.2%)***	***1.35 (0.98, 1.86)***	***0.07***	68/81 (84.0%)	61/85 (71.8%)	1.17 (0.99, 1.38)	0.06	80/81 (98.8%)	82/85 (96.5%)	1.02 (0.98, 1.07)	0.33	760 (449, 1148)	609 (325, 969)
Month 3 *Secondary analysis (Missing = TND)*	45/101 (44.6%)	35/98 (35.7%)	1.25 (0.89, 1.76)	0.21	68/101 (67.3%)	61/98 (62.2%)	1.08 (0.88, 1.33)	0.45	80/101 (79.2%)	82/98 (83.7%)	0.95 (0.83, 1.08)	0.42		
Month 6	13/69 (18.8%)	7/69 (10.1%)	1.86 (0.79, 4.37)	0.16	35/69 (50.7%)	25/69 (36.2%)	1.40 (0.95, 2.07)	0.09	60/69 (87.0%)	54/69 (78.3%)	1.11 (0.95, 1.30)	0.18	409 (79, 621)	325 (95, 548)
Month 12	5/67 (7.5%)	3/62 (4.8%)	1.54 (0.39, 6.19)	0.54	13/67 (19.4%)	11/62 (17.7%)	1.09 (0.53, 2.26)	0.81	39/67 (58.2%)	33/62 (53.2%)	1.09 (0.80, 1.50)	0.57	204 (80, 438)	199 (43, 410)

The median and IQR were calculated among detectable results. All analyses were intention‐to‐treat (ITT) and included all randomized participants other than one participant subsequently found to be HIV positive at enrolment. The primary endpoint was high adherence by tenofovir diphosphate (TFV‐DP) concentration in dried blood spots (DBS), at three months.

^a^ High adherence was defined for M1 as TFV‐DP ≥ 500 fmol/punch; and for months two through 12 as TFV‐DP ≥ 700 fmol/punch.

^b^ Medium adherence was defined for M1 as TFV‐DP ≥ 250 fmol/punch; and for months two through 12 as TFV‐DP ≥ 350 fmol/punch TND denotes target not detected (<16.6 fmol/punch). For the secondary analysis, those who had no DBS measurement, whether due to a missed visit or DBS not being collected at the visit, were categorized as undetectable. The relative risk (RR), confidence interval (CI) and *p*‐value were generated via modified Poisson regression with robust standard errors comparing the outcome by randomized group.

The analysis of high PrEP adherence after Month 3 when the incentives ended in the intervention group, showed a diminishing effect of the intervention. By ITT analysis, the proportions with TFV‐DP ≥ 700 fmol/punch levels were non‐significantly higher in the incentive group: 13 (14%) compared to seven (8%) in the control group at Month 6 (RR = 1.86; 95% CI 0.79, 4.37), and equivalent at Month 12 with five (8%) in the incentive group versus three (5%) in the control group (RR = 1.54, 95% CI 0.38, 6.19).

### 3.5 HIV seroconversions and false‐positive tests

One participant had negative rapid HIV tests at screening and enrolment and a reactive HIV test at one month. Her baseline HIV RNA was positive and thus she was acutely HIV infected at the time of initiating PrEP. She was excluded from post‐baseline analyses, was discontinued from PrEP at Month 1 and referred for HIV treatment. Her genotype resistance assay showed the M184V mutation indicating resistance to emtricitabine, and she was virally suppressed three months after initiation of tenofovir, emtricitabine and efavirenz.

None of the remaining 199 participants seroconverted during 176 person‐years of follow‐up for an HIV incidence of zero (95% CI 0.0, 3.0). Four participants had a total of nine positive dual HIV antigen/antibody tests, which led to short PrEP holds, until HIV RNA testing confirmed that they were not infected.

## 4 DISCUSSION

In this open‐label PrEP demonstration project among 200 young women in a township near Cape Town, South Africa who initiated PrEP, half of the women who attended follow‐up visits had high adherence in the first three months, defined as TFV‐DP ≥ 700 fmol/DBS punch, indicating they took an average of four or more doses per week in the first three months of PrEP use. Notably, all but five samples from the first three months had detectable TFV‐DP, demonstrating some PrEP use in the four to six weeks prior to DSB collection. Just over half persisted with the PrEP programme through 12 months, with 44% attending follow‐up visits and receiving sufficient PrEP refills to have access to PrEP for the entire 12 months of follow‐up and an additional 14% stopping and restarting PrEP for at least some additional time during follow‐up.

Women in both groups received retrospective drug level feedback and counselling about their DBS TFV‐DP levels from the prior four to six weeks; thus, we were not able to determine the effect of drug level feedback alone on PrEP adherence. In a PrEP demonstration project, HPTN 082, which was conducted at the same time as this study, there was no difference in PrEP adherence at six months in the women who received drug level feedback compared to those who received standard adherence support without drug level feedback [13]. All women received drug level feedback, and half were randomized to receive a modest incentive at Months 2, 3 and 4 if they achieved high TFV‐DP levels in the prior month. We observed a non‐statistically significant trend of a higher proportion (approximately one‐third) of women in the intervention group having high adherence at Month 3, although this modest difference did not persist to Month 6 or 12. The study was powered to detect a 22% difference in effect size since it was thought that a relatively large improvement in adherence would be needed to justify the additional costs and logistics of providing conditional incentives. The modest and nonsignificant improvement in adherence in the incentive arm which was observed at Month 3 indicates that additional strategies are needed to overcome early barriers to forming a habit of consistently taking an antiretroviral for HIV prevention, which includes focusing on present rewards [18] or a lack of salience of risks [31]. While some previous studies have demonstrated beneficial impacts of financial incentives on adherence to HIV treatment [32], this is the first study of providing the effect of conditional incentives to young African women initiating PrEP.

Our results highlight the challenges for young South African women related to PrEP adherence execution. PrEP is a novel biomedical HIV prevention and in order to be effective, requires both attendance to clinic visits for refills as well as motivation and ability to take a pill daily. Self‐report of PrEP adherence at Month 1 did not discriminate well in terms of objective TFV‐DP DBS levels, which were used as the primary measure of how well young women took PrEP. Over half persisted with the PrEP programme through 12 months, including the 14% who stopped and restarted PrEP, which is higher than has been observed in other PrEP demonstration projects in young African women [13]. It is plausible that AGYW made decisions about when they needed PrEP and used it episodically in an effective manner, consistent with “prevention effective adherence” in which PrEP adherence is high around times of exposure [33], which is being explored in qualitative interviews among a subset of 3P study participants. The high uptake of PrEP and persistence with the programme through 12 months is encourgaging for a novel HIV prevention method but also highlights the need to improve persistence with daily oral PrEP and to have a choice of methods for those needing less user‐dependent HIV prevention methods.

Given changes in HIV risks and other logistical factors influencing ability to return for visits, PrEP discontinuation should be expected, and understanding the reasons for discontinuation and facilitating restarting are important to incorporate in PrEP programmes. Clinical reasons for holds included side effects, pregnancy due to South African guidelines which restricted PrEP use to non‐pregnant women at the time of the study, and temporary holds due to reactive HIV tests which were subsequently demonstrated to be false‐positive results. Providers need to be prepared for false‐positive screening tests with the dual antigen/antibody test and lower positive predictive value in PrEP users [34, 35]. Most women who stopped PrEP in this study discontinued due to missed visits or based on personal preference, including perceived lower risk, pill fatigue and loss of interest.

No incident HIV infections were observed after PrEP initiation in this population of 200 women who had a high prevalence (30%) of curable STIs, the majority had a partner of unknown HIV status and low condom use. The incidence of curable STIs (chlamydia, gonorrhoea or trichomonas) was 52/100 person‐years in the first six months, indicating that women remained at risk during follow‐up [36]. The lack of HIV infections observed after PrEP initiation in this study can be contrasted to a historical 3% to 4% HIV incidence rates in recent HIV prevention trials with similar populations of young South African women [1, 2, 3]. Additional research is needed to understand the level of protection against HIV among women with imperfect adherence to PrEP.

Limitations of this study include the modest study size which limits the precision of the results. DBS results were not obtained for 34 (17%) of participants at Month 3, primarily due to missed visits. Since it is not possible to measure adherence in those who did not attend visits, they were assumed to not be adherent in our secondary analysis, the results of which did not differ substantially from the intention‐to‐treat analysis. Visit intervals increased to quarterly after the monthly visits in the first three months, which limit interpretation of whether adherence declined due to less frequent visits or stopping drug level feedback and conditional incentives. However, the study was designed with a visit schedule that could be implemented in public programmes, where monthly visits would be burdensome to PrEP users and providers. Our data on temporal patterns of sexual behaviour and PrEP adherence does not allow granular analysis of the timing of sexual exposure and PrEP use to analyse prevention‐effective adherence behaviour. Lastly, without a non‐PrEP control group, it is not possible to attribute whether the lack of incident HIV infections in this study population is due to PrEP or other factors.

## 5 CONCLUSIONS

In summary, this open‐label PrEP study among young women in a South African township demonstrated moderately high PrEP adherence in the first three months and over half persisted with PrEP refills through nine months and the programme through twelve months. The nonsignificant trend towards a greater proportion of the intervention group having high adherence in the first three months of PrEP use may indicate a potential role for nudges or incentives. However, incentives need to have a stronger and more lasting effect on PrEP adherence and persistence than observed in this study in order to be feasible and cost‐effective for implementation. PrEP delivery programmes need to assist women in decision making about PrEP continuation and assist with adherence support for those who want to continue PrEP. In addition, efficacious long‐acting PrEP formulations and less frequent dosing strategies for oral PrEP may offer more options for young African women who find daily dosing challenging.

## COMPETING INTEREST

Dr. Baeten reports grants from NIH, during the conduct of the study; personal fees from Gilead Science, Janssen, Merck, outside the submitted work; Dr. Connie Celum reports grants from NIH and has served as a scientific advisor to Merck and Gilead Science.

## AUTHORS’ CONTRIBUTIONS

CC, MM, AvS, JMB and LGB conceptualized and designed the study. KG, MD, EM, KN and JD acquired the data. GS and KKT analysed the data. CC and LGB wrote the paper. All authors read and approved the final manuscript.
